# Effects of topping on rhizome, and analysis of chemical composition, antioxidant activity and α-amylase and α-glucosidase inhibition of the aerial parts in *Polygonatum cyrtonema*

**DOI:** 10.1371/journal.pone.0287894

**Published:** 2023-11-02

**Authors:** Wenwen Tang, Yuan Chen, Fengxia Guo

**Affiliations:** 1 College of Agronomy, State Key Laboratory of Aridland Crop Science, Gansu Agricultural University, Lanzhou, China; 2 Tongren Polytechnic College, Tongren, China; 3 College of Life Science and Technology, Gansu Agricultural University, Lanzhou, Gansu, China; MGR College, INDIA

## Abstract

*Polygonatum cyrtonema* is a perennial plant, and it has long been used in traditional Chinese medicine for food and medicine. The medicinal part of *P*.*cyrtonema* is the rhizome; however, the aerial part has not been studied. To understand the effect of the topping of aerial parts on the yield and chemical components of rhizomes, as well as the chemical constituents, antioxidant, and *in vitro* hypoglycemic activities of the aerial stem, leave, and flower parts of *P*.*cyrtonema*, the present study was conducted. The results showed that compared to the control (CK) treatment, the topping of the aerial part increased rhizome weight gain coefficient (3.43) and the total saponin content (37.60 mg/g) significantly (*P*<0.01) than the CK treatment. The contents of total phenols and total flavonoids in PCL and PCF were significantly (*P*<0.01) higher than those in rhizomes; however, the polysaccharide content (10.47%) in PCR (whole rhizome) was higher than that in PCS (3.65%), PCL (5.99%), and PCF (4.76%) content. The protein and amino acid contents in PCS, PCL, and PCF were higher than those in rhizomes. The protein and amino acid contents in PCS, PCL, and PCF were higher than those in rhizomes. PCS, PCL, and PCF showed strong antioxidant activity (DPPH, ·OH, ABTS, and FRAP), which were better than traditional medicinal parts (the rhizome).*In vitro* hypoglycemic results showed that PCS, PCL, and PCF had certain inhibitory activities on α-amylase and α-glucosidase (66.25% and 52.81%), which were close to the hypoglycemic activity of rhizomes (67.96% and 52.22%). The leaf extracts also showed better inhibitory activity. To sum up, the topping measures can improve yield and total saponin content of the rhizomes from *P*.*cyrtonema*, which can be applied to improve production. The stems, leaves, and flowers had a much stronger antioxidant and hypoglycemic activities and higher the total polyphenols, flavonoids, proteins, and amino acid content. Therefore, stems, leaves, and flowers of *Polygonatum* can be fully developed according to different needs. they are typically used in animal feed, food storage and cosmetics.

## 1. Introduction

*Polygonati rhizoma* (Huangjing in chinese) is a Traditional Chinese Medicine (TCM) that has been known for its food and medicinal properties [1]. It has the functions of enhancing physical strength, prolonging life and invigorating spleen and kidney [2]. It has a sweet taste and is traditionally known to be eaten as a food. It is a rich source of non-starch polysaccharides and fructooligosaccharides and has no starch content. It is one of the most cost-effective dietary products for diabetics and elderly people. It is expected to become an emerging high-quality crop with great potential [3]. Modern medical research shows that *Polygonati rhizoma* has various health beneficial effects such as enhancing immune function, regulating blood lipids, lowering blood sugar, and preventing cancer [4]. Moreover, it has been developed as an anti-aging health food and sports nutrition [5, 6].

The *Polygonatum cyrtonema*(*P*.*cyrtonema*) is one of the official source plant of *Polygonati rhizoma* included in the 2020 edition of the "Chinese Pharmacopoeia". Since 2015, the *P*.*cyrtonema* planting area has been gradually increased in Zhejiang, Guizhou, Hunan, Jiangxi, Anhui, Guangxi, Sichuan, Hubei, and other Chinese provinces.

At present, the development and utilization of *P*.*cyrtonema* is limited to only rhizomes, whereas many stems, leaves and flowers are wastes with no utilization. Also, to promote the growth of its rhizomes, the aerial parts were removed, which generates a lot of stems, leaves and flowers during topping period of *P*.*cyrtonema*. Furthermore, the above-ground parts of *P*.*cyrtonema* undergo drying and then burn in an open field, which pollutes the environment of the production area and its surroundings. So far, research on *P*.*cyrtonema* has focused on its traditional medicinal part (rhizome) [7–10], and only a few scholars have studied the aerial parts of *P*.*cyrtonema* [11, 12]. These studies clearly postulated that the above-ground parts of *P*.*cyrtonema* could be utilized as food and medicine. Thus, there is an urgent need to study the functional components and physiological activities of the aerial parts (stems, leaves, and flowers) of *P*.*cyrtonema* to provide data for the utilization of these resources.

In the present study, *P*.*cyrtonema* was used as the research object, to determine the effect of topping on the growth of *P*. *cyrtonema* rhizomes, this study also compared the rhizome yields and the contents of polysaccharides, saponins, flavonoids and phenols with and without topping. In addition, the chemical components, antioxidant activity α-amylase and α-glucosidase inhibition of the removing aerial parts (stems, leaves, flowers) were evaluated to provide functional data for the potential development and future utilization of the aerial parts of *P*.*cyrtonema*.

## 2. Materials and methods

### 2.1 Planting experimental area

The field experiments were carried out in the planting base of Zhengda in Songtao County, Guizhou Province, China. The test area is located at 108°35’-109°23’ east longitude, 27°49’-28°30’ north latitude, with an average altitude of about 750 mm asl and an annual average temperature of 16.5°C. The frost-free period is of 293 days, the annual precipitation is 1378.3 mm, the average annual rainy period is 183 days, and the average sunshine time is 1228 h. The soil is sandy, and the soil fertility is medium. The *Phellodendron* saplings were planted at 5m×2m spacing with a tree height of 1. 5–2. 5 m, which has the shading effect in the field.

### 2.2 Plant sample

The roots of *P*.*cyrtonema* of weight between 18–25 g were collected. The stems, leaves and flowers were collected in early May 2021, while the rhizomes were collected in December 2021. The botanical authentication was carried out by Professor Yuyong Liang at Tongren Polytechnic College. A voucher specimen (TRZYHM_1763) was deposited at in the herbal medicine collection of Tongren Polytechnic College. The rhizomes, stems, leaves, and flowers of *P*.*cyrtonema* were washed to remove impurities, dried at 105°C for half an hour, subsequently dried at 60°C, vacuum-packed, and stored at -80°C until analysis.

### 2.3 Instruments and reagents

Instruments including UV-1801 UV-Vis Spectrophotometer (Beijing Ruili Analytical Instrument Co., Ltd.); SB5200DTD Ultrasonic Cleaner (Ningbo Xinzhi Biotechnology Co., Ltd.); CP214 Electronic Balance (Ohaus Instrument Co., Ltd.); and Ultrapure Water Meter (South Korea) Human Company) were used in this study. Diosgenin (batch number: 111539–202102, purity: 98.7%), D-anhydroglucose reference substance (batch number 110833–201908, mass fraction: 99.8%) were all purchased from China Food and Drug Administration Research Institute. Rutin (batch number: B20771, purity: ≥98%), gallic acid (batch number: B20851, purity: ≥98%) were purchased from Shanghai Yuanye Biotechnology Co., Ltd. Folin phenol reagent, and amino acid mixed reference substances were purchased from Sigma reagent company (Sigma-Aldrich). Methanol, ethanol, anhydrous glucose, dimethyl sulfoxide, anthrone, sulfuric acid, sodium nitrite, aluminum nitrate, sodium hydroxide, n-butanol, vanillin, and glacial acetic acid were all analytical grades and purchased from Shanghai Sinopharm Chemical Reagent Co., Ltd.

### 2.4 Topping test method

At the beginning of May 2021, the topping test (Removal of 1/3 the height of the aerial plants)was carried out during the flowering period of the second year of planting *P*.*cyrtonema*. CT treatment (with topping) and CK treatment(without topping) was set. Each treatment was repeated 3 times, and a total of 6 plots were made with 9 m^2^ (3m × 3m) area.

At the end of November 2021, the production was measured per meter square. The "S" type sampling method was adopted in the plot, and 15 plants were sampled in each plot. According to the fresh rhizomes weight of the sampled plants, the weight gain coefficient of the rhizomes was calculated, and the yield per unit area was measured. The rhizomes of the current year were cut off, and dried at 60°C to determine the rhizomes weight of *P*.*cyrtonema* in the current year under different treatments. Rhizome weight gain coefficient was determined as the equation (Eq 1):

$$\text{Cg}=\left(\text{Wp}-\text{Ws}\right)/\text{Ws}$$ (1)

where Cg is the Rhizome weight gain coefficient, Wp is the fresh weight of rhizome per plant, Ws is the fresh weight of seed root.

### 2.5 Phytochemical analysis

The polysaccharide content was determined as per the 2020 edition of "Chinese Pharmacopoeia" [2], using the anthrone-sulfuric acid chromogenic method. The polysaccharide content was determined at 582 nm wavelength using anhydrous glucose as reference (y = 5.1307x + 0.0064; R^2^ = 0.9993).

Total saponins content (mg/g) was determined according to the vanillin-glacial acetic acid-perchloric acid colorimetric method [13], with minor modifications, 1.0 g of each sample powder was mixed with 30 mL of 80% ethanol and extracted using an ultrasonic bath at 25°C for 30 min (3 times). The absorbance was measured at 532 nm, Methanol as blank control was used to draw standard curve with diosgenin as reference (y = 6.2371x − 0.0055; R^2^ = 0.9997).

The total polyphenol content (mg/g) was determined according to Folin’s phenol colorimetric method [14]. A standard curve was drawn with gallic acid as reference (y = 3.7514x + 0.006; R^2^ = 0.9991), and the total polyphenol content in the sample was calculated.

The total flavonoids content (mg/g) was determined by the NaNO_2_-Al(NO_3_)_3_-NaOH colorimetric method [15]. A standard curve was drawn with rutin as the reference (y = 10.39x+0.0014; R^2^ = 0.9998), and the total flavonoid content in the sample was calculated.

The Kjeldahl method was used to determine the protein content [16]. The protein content is the nitrogen content multiplied by 6.25.

The determination of amino acids was completed by SykamS433 amino acid analyzer (Germany), referring to GB 5009.124–2016 "Determination of Amino Acids in Food Safety National Standard" (China) [17].

### 2.6 Antioxidant activity analysis

#### 2.6.1 Extract preparation

To 1.0 g of *P*.*cyrtonema* rhizome, stem, leaf, and flower sample powder, respectively, add 50 mL of 50% ethanol. The mixture was ultrasonicated for 10 min and then heated under reflux for 1 h, filtered, and transferred to a 50 mL volumetric flask. The volume was makeup using 50% ethanol.

#### 2.6.2 DPPH scavenging assay

Each sample was diluted into solutions of different concentrations, and 2 mL of each diluted solution was mixed with 2.0 mL of DPPH methanol solution. The absorbance was measured at 517 nm [18], and vitamin C (Vc) was considered as the positive control. The scavenging rate of DPPH free radicals for each sample was calculated using the equation (Eq 2):

$$\text{Y}\left(\%\right)=100*\left[{\text{A}}_{0}-\left(\text{As}-{\text{A}}_{\text{S}0}\right)\right]/{\text{A}}_{0}$$ (2)

where Y (%) is the scavenging rate of DPPH free radicals, A_0_ is the absorbance value of the blank group, As is the absorbance value of different samples, and A_S0_ is the absorbance value of the sample itself. According to the clearance rate of DPPH for each sample at different concentrations, IC_50_ was calculated using the probability regression analysis of SPSS.

#### 2.6.3 ABTS scavenging assay

To prepare the working solution, the ABTS radical solution was prepared by mixing 10.0 mL of 7.0 mM ABTS and 5.0 mL of 7.0 mM potassium persulfate. The mixture was maintained at room temperature for 12 h in the dark to allow ABTS^·+^ formation before being diluted with ethanol to an absorbance of 0.700 ± 0.02 at 734 nm. 2 mL of ABTS^·+^ was added to 50 μL of each sample solution at different concentrations and left for 6 min in the dark at room temperature. The absorbance was measured at 734 nm [19], with Vc as a positive control. The scavenging rate of the sample to ABTS free radicals was calculated following the equation (Eq 3):

$$\text{T}\left(\%\right)=100*\left[\left({\text{A}}_{0}-{\text{A}}_{\text{S}}\right)/{\text{A}}_{0}\right]$$ (3)

where T (%) is the ABTS free radical scavenging rate, A_0_ is the absorbance value of the blank group, and As is the absorbance value of different samples. According to the clearance rate of ABTS of each sample at different concentrations, IC_50_ was calculated using the probability regression analysis of SPSS.

#### 2.6.4·OH scavenging assay

To 1.5 mL of each sample solution with different concentrations, 1.0 mL of 2.5 mmol/L salicylic acid solution, 1.0 mL of 5 mmol/L FeSO_4_ solution, and 2.0 mL of distilled water were added and mixed well. To this solution, 1.0 mL of 5 mmol/L H_2_O_2_ was added. The mixture was placed at a constant temperature water bath at 37°C for 30 min, and the absorbance was measured at a wavelength of 510 nm, with V_C_ as a positive control. The scavenging rate of·OH radicals was calculated according to the equation (Eq 4) [20]:

$$\cdot \text{OH}\left(\%\right)=100*\left[{\text{A}}_{0}-\left({\text{A}}_{2}-{\text{A}}_{1}\right)\right]/{\text{A}}_{0}$$ (4)

where·OH (%) is the scavenging rate of ·OH radicals, A_0_ is the absorbance of the blank control; A_2_ is the absorbance of the sample solution with H_2_O_2_; A_1_ is the absorbance of the sample solution without H_2_O_2_.

#### 2.6.5 FRAP reducing power

Referring to the method of Wang et al. [21], 0.5 mL of each sample solution after proper dilution was mixed with 5 mL of FRAP working solution (prepared for current use) in a water bath at 37°C. After 30 min, the absorbance of the reaction solution was measured at 593 nm. The standard curve was drawn with different concentrations of V_C_ solutions (y = 0.0024x + 0.1269 R^2^ = 0.9992), and the total reducing power of each sample was represented by the equivalent content of V_C_ (unit: μg/mL V_C_ eq/gsample).

### 2.7 α-amylase and α-glucosidase inhibition assay

To 10 g of rhizomes, stems, leaves and flowers sample powder, 250 mL of 50% ethanol was added. The mixture was ultrasonicated for 30 minutes, heated, and refluxed in a water bath for 1 h. The extract was vacuum evaporated and concentrated to dryness. The inhibition rates of α-glucosidase and α-amylase were determined, and acarbose was used as a positive control.

#### 2.7.1 α-glucosidase inhibitory activity

The α-glucosidase inhibitory activity was determined according to the method of Tiara da et al. [22]. The extracts of each sample were dissolved in 50% DMSO. The samples solution of different concentrations were mixed with α-glucosidase (0.5 U/mL) and phosphate buffer (0.1 M, pH 6.8) and kept at 37°C for 10 min in a constant temperature water bath. The PNPG solution (5 mmol/L) was used to start the reaction. After 10 min at 37°C, 400 μL of Na_2_CO_3_ (0.2 mol/L) was added immediately to stop the reaction, and the absorbance was measured at 405 nm. The inhibitory effect of α-glucosidase was calculated according to the equation (Eq 5)

$$\text{inhibition}\left(\%\right)=100*\left[{\text{A}}_{0}-\left({\text{A}}_{1}-{\text{A}}_{2}\right)/{\text{A}}_{0}\right]$$ (5)

where A_0_ is the absorbance of the control group, A_1_ is the absorbance of the sample group, and A_2_ is the absorbance of the blank group.

#### 2.7.2 α-amylase inhibitory activity

The DNS (3,5-dinitrosalicylic acid) colorimetric method was used to determine the α-amylase inhibitory activity [23]. Briefly, each sample extract was dissolved with 50% DMSO, and the sample solution of different concentrations was mixed with α-amylase (10 U/mL) and phosphate buffer (0.1 M, pH 6.8). The reaction was performed at a constant temperature water bath (37°C) for 10 min, and 1% starch solution was added to continue the reaction for 5 min. Then 500 μL of DNS reagent was added, and the sample was kept in a boiling water bath for 5 min. After appropriate dilution, the absorbance was measured at 540 nm. The α-amylase inhibitory effect was calculated according to the equation (Eq 5).

$$\text{inhibition}\left(\%\right)=100*\left[{\text{A}}_{0}-\left({\text{A}}_{1}-{\text{A}}_{2}\right)/{\text{A}}_{0}\right]$$ (5)

where A_0_ is the absorbance of the control group, A_1_ is the absorbance of the sample group, and A_2_ is the blank group absorbance.

### 2.8 Data analysis

Significant differences between mean values were determined using one-way ANOVA at different significance levels. In addition, one-way ANOVA and determinations of the IC_50_ values for DPPH and ABTS radical scavenging activity were performed using SPSS software V. 19.0. All experiments were conducted in triplicate, and IC_50_ values were determined by probit regression analysis.

## 3. Results and discussion

### 3.1 Effects of plant topping on rhizome of *P*.*cyrtonema*

The rhizome yield and polysaccharide, total saponins, total flavonoids, and total polyphenols contents of topping and non-topping of samples are shown in Table 1. From the data in Table 1, topping *P*.*cyrtonema* has a significant impact on the fresh weight of the rhizome per plant and the yield per unit area. Furthermore, it was found that the rhizome weight gain coefficient can more accurately reflect the effect of topping on the rhizome weight gain of *P*.*cyrtonema*. These data showed that the topping of *P*.*cyrtonema* during the flowering period can significantly increase the yield of rhizomes. Removing some leaves and flowers Removing flowers can reduce the nutrient consumption of rhizome [24, 25]. On the other hand, it may be possible to reduce the nutrient consumption of the aerial part of *P*.*cyrtonema* by topping so that the rhizome can distribute more nutrients and promote its expansion. The wound-inducing effect after the topping causes the strong compensatory growth of *P*.*cyrtonema* [26, 27], thereby driving the accumulation of rhizome biomass.

**Table 1:** Effects of plant topping on rhizome yield and components of *P*. *cyrtonema*.

treatments	The fresh rhizome weight gain coefficient	the yield per unit area (kg/m2)	polysaccharide(%)	total saponins (mg/g)	total polyphenols (mg/g)	total flavonoids (mg/g)
CT	3.43^Aa^	1.17^Aa^	8.80±0.39^Aa^	37.60±2.91^Aa^	7.51±0.48^Ab^	1.08±0.11^Bb^
CK	2.63^Bb^	0.93^Bb^	8.64±0.30^Aa^	32.53±2.67^Bb^	7.94±037^Aa^	1.42±0.15^Aa^

The data are presented as the means ± SD. Within each column, the different superscripted small and capital letters indicate significant and highly significant differences at *P* < 0.05 and *P*< 0.01, respectively, based on ANOVA-Duncan multiple comparison results. CT, with toppin; CK, without topping.

The output of Chinese herbal medicines is related to its economic value, and the content of the medicinal components is a decisive factor for the quality of Chinese herbal medicines. The rhizomes of *P*.*cyrtonema* are similar to bamboo whips, aerial parts grow on the current year rhizomes. Therefore, the effect of topping on the quality of rhizomes was determined, and the functional components of the rhizomes of the current year correspond to the plants with and without topping. From the data in Table 1, With and without topping, there is no significant difference in polysaccharide content, and both meet the requirement of "2020 Pharmacopoeia" [2]. The saponin content of the rhizome of topping was 37.60 mg/g, which was significantly higher (*P* < 0.01) than that of the rhizome without topping (32.53 mg/g). Topping can significantly increase the content of total saponins, and saponins showed anti-inflammatory, hypoglycemic, intestinal flora regulation, antidepressant and other pharmacological activities [9, 28]. The content of total polyphenols and total flavonoids in Polygonatum rhizomes of topping (7.51 mg/g, 1.08 mg/g) were lower than those without topping (7.94 mg/g, 1.42 mg/g). Light exposure should increase the content and activity of flavonoid synthases such as phenylalanine ammonia-lyase, chalcone isomerase, flavanone-3-hydroxylase, and flavonol synthase [29]. Light played a major role in increasing the flavonoids content in plants. After topping, the aboveground plants receive less light, thus decreasing the content of flavonoids in *Polygonatum cyrtonema*. Most flavonoids contain phenolic hydroxyl group (·OH), an important part of polyphenols. The decrease in the flavonoids content after topping is also one of the reasons for the decrease in the polyphenols content. These metabolites may be synthesized in the stems and then transferred to the roots or directly synthesized in the roots. The rhizomes’ physiological and secondary metabolic mechanisms in response to light are still unclear, and further research is needed. Although the content of flavonoids and polyphenols in rhizomes decreased after decapitation, however, Polysaccharide and saponins are the main active components of rhizomes of *P*.*cyrtonema*, and they are commonly used to identify the quality of *Polygonati rhizoma* [30–32]. As a result, the content and yield of polysaccharides increased significantly. However, further research needs to be conducted to determine the regulatory mechanism of the distribution of nutrients in the aboveground and underground parts of *P*.*cyrtonema* to accumulate to the roots.

### 3.2 Chemical components of aerial parts in *P*.*cyrtonema*

The contents of polysaccharides, total saponins, total phenols, total flavonoids and proteins in each sample are shown in Table 2. The data in Table 2 showed that the rhizomes (PCR, the whole rhizomes), stems (PCS), leaves (PCL), and flowers (PCF) of *P*.*cyrtonema* contain functional components such as polysaccharides, total saponins, total phenols, total flavonoids, and proteins; However, their concentrations were significantly different. For instance, the polysaccharide content in PCR was the highest (10.47%), followed by leaves (5.99%) and flowers (4.76%), while the polysaccharide content in the stem was the lowest (3.65%). The content of total saponins in flowers (36.68mg/g) is very close to the rhizomes (39.09mg/g) content, and there is no significant difference between the two contents. The content of total saponins in stems and leaves is lower and represents only 1/7 of that in flowers (~1/5). The contents of total phenols and total flavonoids in different samples were consistent, all of which were in the order of PCF>PCL>PCS>PCR, which was inconsistent with the study of Zhao et al. [33]. The contents of total phenols and flavonoids in flowers, leaves, and stems were significantly (P<0.01) higher than those in rhizomes, especially the polyphenols content in flowers was much higher than that in rhizomes. The results were consistent with Zhang et al. [12], which could be related to the higher content of anthocyanins in flowers. The protein content in the rhizome was the lowest (3.08%), which was only half of the protein content in the stem. The protein content in the leaf and flower was higher, 13.81% and 11.64%, respectively, and about 3.7–4.5 times in the rhizome. These data showed that the non-medicinal parts such as stems, leaves and flowers contain more bioactive compounds than the rhizomes. The content of total phenols, total flavonoids and protein in the leaves is high, and it has a good potential to be developed into functional food and skincare products.

**Table 2:** Polysaccharide (POL), total saponins(TS), total polyphenol (TP), total flavonoid (TF), and protein contents.

Samples	POL(%)	TS (mg/g)	TP (mg/g)	TF (mg/g)	Protein (%)
PCS	3.65±0.28^Dd^	4.99±0.20^Bb^	21.52±0.43^Cc^	2.62±0.15^Cc^	9.87±0.79^Cc^
PCL	5.99±0.22^Bb^	7.11±0.17^Bb^	37.57±1.12^Bb^	3.09±0.12^Bb^	13.81±0.91^Aa^
PCF	4.76±0.17^Cc^	36.68±2.53^Aa^	53.83±2.33^Aa^	4.48±0.13^Aa^	11.64±0.70^Bb^
PCR	10.47±0.47^Aa^	39.09±1.37^Aa^	9.63±0.56^Cc^	1.64±0.09^Dd^	3.08±0.33^Dd^

The data are presented as the means ± SD. PCR, *P*. *cyrtonema* rhizome; PCS, *P*. *cyrtonema* stem;

PCL, *P*. *cyrtonema* leaves; PCF, *P*.*cyrtonema* flower.

Within each column, the different superscripted small and capital letters indicate significant and highly significant differences at *P*< 0.05 and *P* < 0.01, respectively, based on ANOVA-Duncan multiple comparison results.

The data in Table 3 showed the total amino acids, their content, and individual content in rhizomes (PCR), stems (PCS), leaves (PCL) and flowers (PCF). *P*.*cyrtonema* stems, leaves, and flowers are rich in amino acids, which are much higher than rhizomes. The total amino acid content in leaves is the highest (23.25%), about 3.16 times that in the rhizomes. The contents of valine, leucine, isoleucine, phenylalanine and lysine in the stems, leaves and flowers are all higher, especially in the leaves; the contents of these essential amino acids are above 1%. These amino acids provide better health care functions such as regulating blood sugar and improving immunity. The contents of umami amino acids such as aspartic acid and glutamic acid are higher in the leaves. These results indicated that stems (PCS), leaves (PCL) and flowers (PCF) contained more abundant amino acids than rhizomes. This should be taken seriously in the development and utilization of the aboveground part of *P*.*cyrtonema*.

**Table 3:** Amino acid contents of PCR, PCS, PCL, and PCF.

Amino acid	Amino acid content (%)			
	PCS	PC L	PCF	PCR
Asp	1.93	2.42	1.13	1.57
Thr	0.94	1.08	0.43	0.25
Ser	0.96	1.11	0.5	0.37
Glu	3.45	4.52	2.72	1.36
Gly	0.98	1.10	0.67	0.29
Ala	1.14	1.30	1.00	0.23
Val	1.29	1.54	0.83	0.31
Met	0.28	0.32	0.06	0.05
Ile	0.85	1.02	0.56	0.14
Leu	1.82	2.02	0.98	0.56
Tyr	1.04	1.19	1.02	0.77
Phe	1.12	1.25	0.69	0.23
Lys	1.03	1.45	0.78	0.28
His	0.46	0.60	0.26	0.14
Arg	1.12	1.32	0.87	0.67
Pro	0.86	1.01	0.58	0.13
Total content	19.27	23.25	13.08	7.35

### 3.3 Antioxidant activity of aerial parts in *P*.*cyrtonema*

Antioxidants can be divided into synthetic antioxidants and natural antioxidants. With the research on the toxicology of synthetic antioxidants and the enhancement of people’s health awareness, the development of natural antioxidants came to light. Most natural antioxidants are obtained from plants [34]. Intake of natural antioxidants Many studies have shown that the natural antioxidant components contained in plants can effectively reduce the incidence of aging-related diseases such as cardiovascular disease, diabetes, cancer, etc. by scavenging free radicals [35, 36].

**Fig 1:**
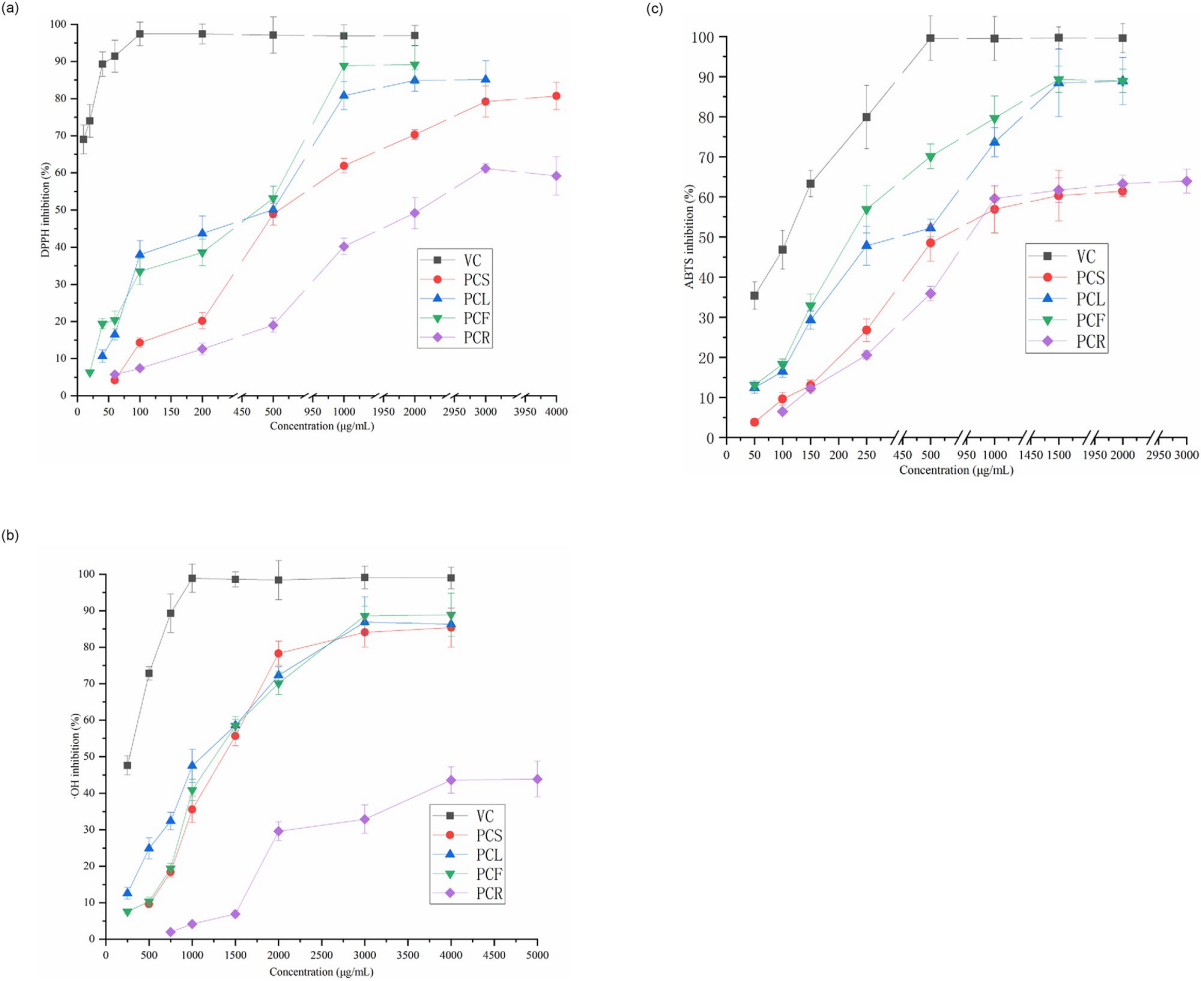
**A**. Inhibition of DPPH radical for samples. **B**. Inhibition of·OH radical for samples. **C**. Inhibition of ABTS radical for samples.

DPPH is a purple, very stable free radical, which is used to measure the ability of various antioxidants to scavenge free radicals. The scavenging effect on DPPH free radicals is shown in Fig 1A. Both leaves and flowers have high DPPH free radical scavenging ability and the scavenging rate increases with increasing concentration. Among them, flowers have the strongest scavenging ability to DPPH free radicals, and at 1000 μg/mL, the scavenging rate reaches 88.9%, respectively. This could be related to the high polyphenols and flavonoids concentration in the flowers. In the case of stems and leaves samples at 3000 μg/mL, the scavenging abilities of DPPH free radicals were 79.2% and 85.1%, respectively, which were higher than those of rhizomes (61.2%). This may be related to the fact that stems and leaves contain more flavonoids and polyphenols than rhizomes and can directly capture free radicals.

As shown in Fig 1B, the stems, leaves, and flowers of *P*.*cyrtonema* also showed comparatively stronger ·OH radicals scavenging ability than rhizomes. For example, at a concentration of 4000 μg/mL, the·OH radicals scavenging abilities of the stems, leaves, and flowers are very close, reaching more than 85%, while the scavenging power of rhizomes is only 43.6%. However, at lower concentrations (250–1000 μg/mL), the leaves showed stronger ·OH radical scavenging ability than stems and flowers. Moreover, the scavenging ability of flowers changed greatly with increasing concentration, indicating that the flower has a strong ability to scavenge ·OH radicals.

The scavenging effect on ABTS free radicals is shown in Fig 1C. Similar to the results of DPPH and ·OH free radical scavenging experiments, flowers and leaves showed higher scavenging ability to ABTS free radicals at a concentration of 1500 μg/mL. At this concentration, the ABTS clearance rates of leaf and flower extracts reached 88.4% and 89.3%, respectively, while the ABTS clearance rates of stems at this concentration are weaker (60.3%), like those of rhizomes.

The IC_50_ values of the stem, leaf and flower extracts for scavenging DPPH, ·OH, and ABTS free radicals, and the FRAP total reducing power analysis results are shown in Table 4. The smaller the IC_50_, the stronger the free radical scavenging ability. It can be seen from Table 4 that for the IC_50_ of DPPH and ABTS, the scavenging effect of stems, leaves, and flowers is stronger than that of rhizomes (p<0.01), but compared with stems (719.35, 862.39 μg/mL), leaves (301.86, 342.87 μg/mL) and flower (251.44, 264.61 μg/mL) showed stronger DPPH and ABTS free radical scavenging effects (*P*< 0.01). However, unlike DPPH, the IC_50_ of leaf against·OH (1069.93 μg/mL) was the smallest, indicating that leaves had a stronger ability to scavenge ·OH, followed by flowers and stems.

**Table 4:** IC_50_ values of DPPH, ·OH and ABTS radicals inhibition and test results of FRAP assays of extracts of rhizome, stem, leaf and flower.

Samples	DPPH IC_50_ values (μg/mL)	·OH IC_50_ values (μg/mL)	ABTS IC_50_ values (μg/mL)	FRAP assays result (μg/mL V_C_ eq/gsample)
PCS	719.35±6.06^Bb^	1348.63±38.89^Aa^	862.39±26.04^Bb^	552.69±12.99^Cc^
PCL	301.86±9.48^Cc^	1069.93±54.89^Cc^	342.87±9.21^Cc^	1232.43±17.52^Bb^
PCF	251.44±2.19^Dd^	1280.97±16.60^Bb^	264.61±18.42^Dd^	1343.65±49.65^Aa^
PCR	2069.67±26.53^Aa^	ND	1039.87±25.13^Aa^	307.85±3.46^Dd^

Note:The data are presented as the means ± SD. ND, not detected; Within each column, the different superscripted small and capital letters in indicate significant and highly significant differences at *P*< 0.05 and *P* < 0.01, respectively, based on ANOVA-Duncan multiple comparison results.

The reducing power is also an important indicator of measuring antioxidant activity. As shown in Table 4, the total reduction of FRAP scavenging power was similar to that of DPPH and ABTS, with higher reducing power for flowers (1343.65 μg/mL V_C_ eq/gsample), leaves (1232.43 μg/mL V_C_ eq/gsample), and stems (552.69 μg/mL V_C_ eq/gsample), but both were significantly stronger (*P*< 0.01) than the reducing power of rhizomes.

The above results of DPPH, ·OH, ABTS and FRAP showed that the stems, leaves, and flowers of *P*.*cyrtonema* have strong free radical scavenging ability and antioxidant activity. The inhibitory activity of the rhizomes was significantly lower than other samples (*P* < 0.01), Researchers found that the antioxidant and nitrite scavenging capacity appeared to be mainly influenced by the flavonoids and polyphenols, and positively relevant to their contents [37]. Natural plants, vegetables, and fruits are rich in a variety of antioxidant substances, such as phenols and flavonoids, which can improve the SOD (superoxide dismutase) and catalase activity in the body and inhibit the formation of nitrite and nitrosamines [38]. While the phenols, and flavonoids content in the aerial parts (leaves and flowers) are higher, which should be an important reason for its higher antioxidant activity. These also indicate that the aerial parts (leaves and flowers) has a stronger anti-aging effect and could be comprehensively utilized in health-promoting effects, and the aerial parts (leaves and flowers) is more suitable as a high-efficiency and low-toxic natural antioxidant than the rhizomes. In addition, the antioxidant activities of stems, leaves, and flowers in the oxidation system were lower than Vc, which may be because the content of active antioxidant substances in the crude extracts of stems, leaves, and flowers are not high enough.

In addition, there are differences in the free radical scavenging ability of DPPH, ·OH, ABTS, and the antioxidant capacity of FRAP among the stems, leaves, and flowers of *Polygonum cyrtonema*, which could be related to the different mechanisms of different antioxidant methods. The antioxidant activities of stems, leaves, and flowers in the oxidation system were lower than Vc, which may be because the content of active antioxidant substances in the crude extracts of stems, leaves, and flowers are not high enough.

### 3.4 α-amylase and α-glucosidase inhibition of aerial parts from *P*.*cyrtonema*

Modern research showed that Rhizoma Polygonati has the effect of regulating blood sugar [28], and α-glucosidase and α-amylase are the key enzymes involved in the digestion and degradation of carbohydrates in the small intestine. Therefore, limiting the enzymatic activity can slow down the starch breakdown and lower postprandial blood sugar levels in people with diabetes. In this study, the hypoglycemic activities were evaluated by measuring the α-glucosidase and α-amylase activity inhibition by the aerial part of the stem, leaf, and flower extracts of *P*. *cyrtonema*. Table 5 shows the inhibition rates and IC_50_ values of α-glucosidase and α-amylase by the *Polygonatum* rhizomes, stems, leaves and flowers extracts at the same concentration. The results showed that at 3000 μg/mL, the stems, leaves and flowers of *P*.*cyrtonema* showed inhibitory activity against α-glucosidase and α-amylase. The inhibition rates of α-amylase and α-glucosidase were 66.25% and 52.81% for flower extracts, close to rhizomes (67.96% and 52.22%), and the leaf extract also showed better inhibitory activity. The lowest inhibitory activity of stem was only 32.23% and 18.53%. The IC_50_ values of α-amylase and α-glucosidase of flowers are lower, followed by the rhizomes. In addition, studies have shown that the inhibitory activity of each sample on α-amylase is stronger than that on α-glucosidase. At the same concentration, the inhibition rate of the same sample on α-amylase is greater than that of α-glucosidase.

**Table 5:** Inhibition and C_50_ values of the α-amylase and α-glucosidase activities of extracts of rhizome, stem, leaf and flower.

Samples	α-amylase		α-glucosidase	
	Inhibition(%)	IC_50_ values (μg/mL)	Inhibition(%)	IC_50_ values (μg/mL)
PCS	32.23±1.44^Cd^	4746.58±55.05^Aa^	18.53±0.47^Cc^	ND
PCL	56.83±4.48^Bc^	2660.09±77.24^Bb^	46.31±1.07^Bb^	3406.31±47.75^Aa^
PCF	66.25±1.08^Ab^	1681.25±16.72^Cc^	52.81±1.75^Aa^	2666.73±34.72^Bb^
PCR	67.96±0.94^Aa^	1525.24±28.91^Dd^	52.22±0.57^Aa^	2369.04±36.56^Cc^

Note:The data are presented as the means ± SD. ND, not detected; Within each column, the different superscripted small and capital letters in indicate significant and highly significant differences at *p* < 0.05 and *p* < 0.01, respectively, based on ANOVA-Duncan multiple comparison results.

Percent of inhibition (%), concentration of extractswas3000μg/mL.

To sum up, the aerial parts of *P*.*cyrtonema* have certain inhibitory activity on α-amylase and α-glucosidase, among which the flower extract has the strongest inhibitory effect, and the stem showed the lowest, which could be related to the higher content of saponins, polyphenols, and flavonoids in flowers. Polyphenols and flavonoids can regulate blood sugar by promoting insulin synthesis and secretion and inhibiting glucose uptake and transport [39, 40].

## 4. Conclusions

This study firstly integrated factors such as rhizome yield, rhizome weight gain coefficient, and the content of bioactive components, and postulated that topping effectively increased the cultivation yield of *P*.*cyrtonema*. Furthermore, the results of *in vitro* evaluation of the health-promoting abilities of stems, leaves and flowers of *P*.*cyrtonema* such as antioxidant and α-amylase and α-glucosidase inhibition, also proved that these parts have health beneficial activities, and the antioxidant effects of leaves and flowers are far better than those of rhizomes and flowers. Also, the study showed that the above-ground parts of *P*.*cyrtonema* have similar α-amylase and α-glucosidase inhibitory ability as rhizomes. In addition, compared with the rhizomes, the aerial parts of *P*.*cyrtonema* have the characteristics of faster growth, strong regeneration ability, and large biological yield. So, they have broader development potential and application value. To develop these parts of *P*.*cyrtonema* into a more widely used health product, further research is needed on the antibacterial and anti-fatigue pharmacological activities and long-term toxicological experiments.

## Supporting information

S1 File (ZIP)
