# Supplementary material for: Effects of topping on rhizome, and analysis of chemical composition, antioxidant activity and α-amylase and α-glucosidase inhibition of the aerial parts in Polygonatum cyrtonema
Source: PLoS One. 2023 Nov 2;18(11):e0287894. doi: 10.1371/journal.pone.0287894 (PMC10621978; doi:10.1371/journal.pone.0287894)
Supplement: S1 File — (ZIP) [file pone.0287894.s001.zip › raw dataú¿Huangjingú⌐/AA-PCF.pdf]

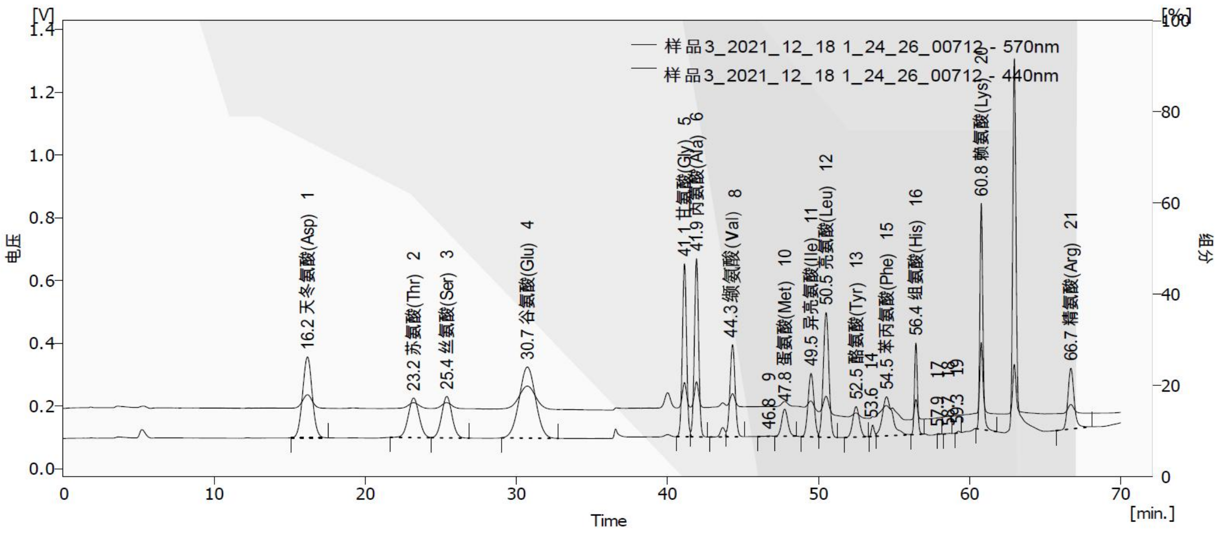

	名称	保留时间[min]	含量<br>[%]	分离度<br>[-]
1	天冬氨酸(Asp)	16.165	1.127	
2	苏氨酸(Thr)	23.195	0.425	5.655
3	丝氨酸(Ser)	25.387	0.499	1.705
4	谷氨酸(Glu)	30.725	2.719	3.277
5	甘氨酸(Gly)	41.123	0.668	8.260
6	丙氨酸(Ala)	41.920	1.002	1.541
8	缬氨酸(Val)	44.301	0.831	1.020
10	蛋氨酸(Met)	47.757	0.060	1.276
11	异亮氨酸(I le)	49.501	0.560	2.346
12	亮氨酸(Leu)	50.496	0.983	1.388
13	酪氨酸(Tyr)	52.483	1.019	2.616
15	苯丙氨酸(Phe)	54.499	0.687	1.177
16	组氨酸(His)	56.440	0.783	2.469
20	赖氨酸(Lys)	60.768	0.261	4.409
21	精氨酸(Arg)	66.701	0.870	10.985

	名称	保留时间[min]	含量<br>[%]	分离度<br>[-]
1	脯氨酸(Pro))	40.008	0.576	
