# Supplementary material for: Effects of topping on rhizome, and analysis of chemical composition, antioxidant activity and α-amylase and α-glucosidase inhibition of the aerial parts in Polygonatum cyrtonema
Source: PLoS One. 2023 Nov 2;18(11):e0287894. doi: 10.1371/journal.pone.0287894 (PMC10621978; doi:10.1371/journal.pone.0287894)
Supplement: S1 File — (ZIP) [file pone.0287894.s001.zip › raw dataú¿Huangjingú⌐/AA-PCL.pdf]

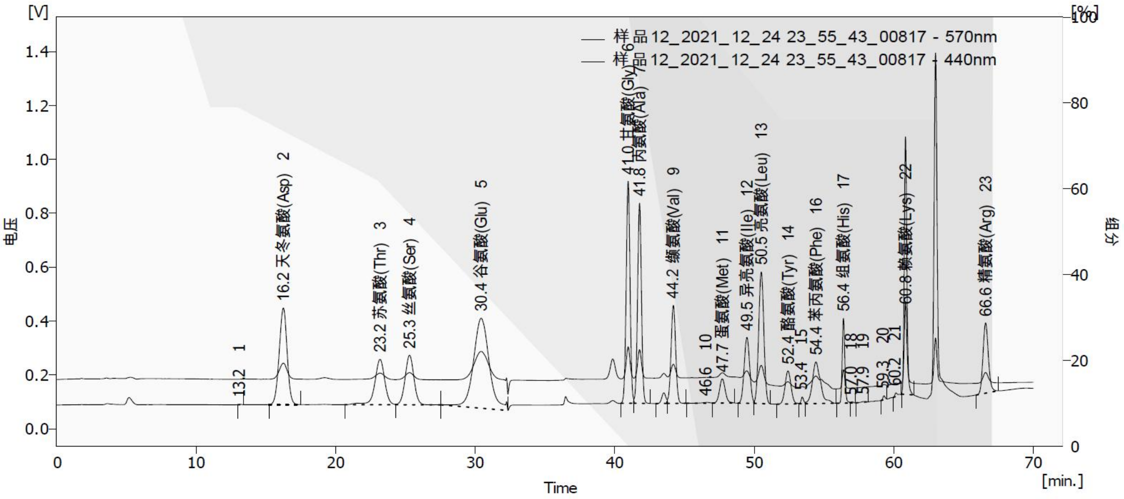

	名称	保留时间[min]	含量<br>[%]	分离度<br>[-]
1	天冬氨酸(Asp)	16.245	2.416	4.524
2	苏氨酸(Thr)	23.176	1.079	6.121
3	丝氨酸(Ser)	25.288	1.109	1.780
4	谷氨酸(Glu)	30.421	4.520	3.306
5	甘氨酸(Gly)	40.955	1.102	8.552
6	丙氨酸(Ala)	41.776	1.299	1.615
8	缬氨酸(Val)	44.195	1.538	1.153
10	蛋氨酸(Met)	47.707	0.323	1.301
11	异亮氨酸(I le)	49.472	1.017	2.367
12	亮氨酸(Leu)	50.496	2.021	1.425
13	酪氨酸(Tyr)	52.413	1.185	2.579
15	苯丙氨酸(Phe)	54.416	1.245	1.415
16	组氨酸(His)	56.387	1.447	2.831
20	赖氨酸(Lys)	60.835	0.601	1.281
21	精氨酸(Arg)	66.576	1.319	11.094

	名称	保留时间[min]	含量<br>[%]	分离度<br>[-]
1	脯氨酸(Pro))	39.856	1.009	
