# Supplementary material for: Effects of topping on rhizome, and analysis of chemical composition, antioxidant activity and α-amylase and α-glucosidase inhibition of the aerial parts in Polygonatum cyrtonema
Source: PLoS One. 2023 Nov 2;18(11):e0287894. doi: 10.1371/journal.pone.0287894 (PMC10621978; doi:10.1371/journal.pone.0287894)
Supplement: S1 File — (ZIP) [file pone.0287894.s001.zip › raw dataú¿Huangjingú⌐/AA-PCR.pdf]

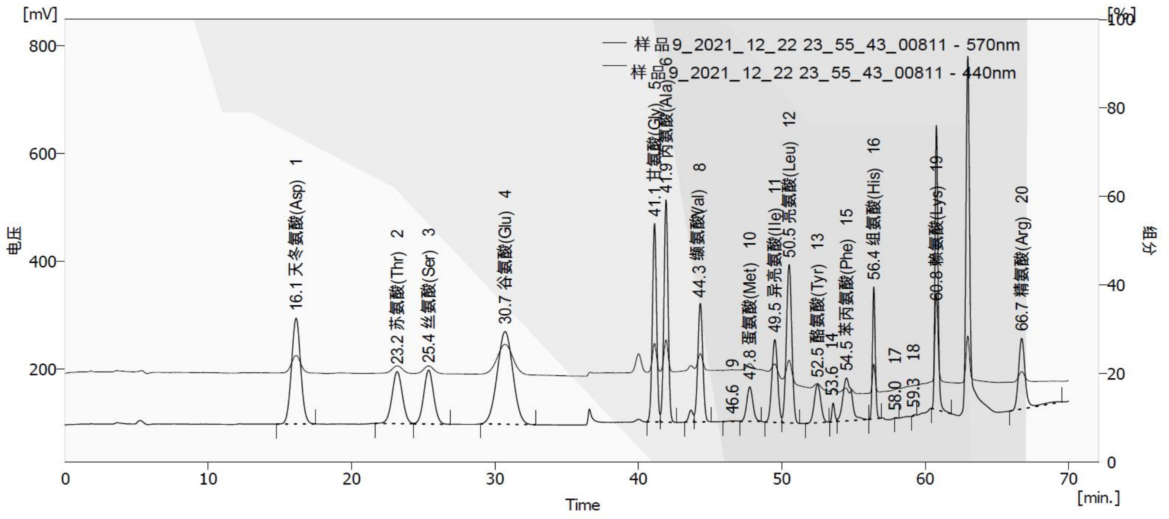

	名称	保留时间[min]	含量<br>[%]	分离度<br>[-]
1	天冬氨酸(Asp)	16.131	1.574	
2	苏氨酸(Thr)	23.179	0.253	5.629
3	丝氨酸(Ser)	25.373	0.369	1.695
4	谷氨酸(Glu)	30.704	1.355	3.236
5	甘氨酸(Gly)	41.120	0.287	8.115
6	丙氨酸(Ala)	41.923	0.231	1.492
8	缬氨酸(Val)	44.312	0.308	1.021
10	蛋氨酸(Met)	47.760	0.050	1.046
11	异亮氨酸(I le)	49.507	0.141	2.273
12	亮氨酸(Leu)	50.507	0.560	1.362
13	酪氨酸(Tyr)	52.488	0.773	2.541
15	苯丙氨酸(Phe)	54.504	0.227	1.211
16	组氨酸(His)	56.411	0.282	2.481
20	赖氨酸(Lys)	60.779	0.140	3.994
21	精氨酸(Arg)	66.725	0.666	10.005

	名称	保留时间[min]	含量<br>[%]	分离度<br>[-]
1	脯氨酸(Pro))	40.011	0.130	
